# A First Clinical Trial on Botulinum Toxin-A for Chronic Muscle-Related Pain in Cerebral Palsy

**DOI:** 10.3389/fneur.2021.696218

**Published:** 2021-08-16

**Authors:** Dan Jacobson, Kristina Löwing, Kjell Kullander, Britt-Marie Rydh, Kristina Tedroff

**Affiliations:** ^1^Neuropediatric Unit, Department of Women's and Children's Health, Karolinska Institutet, Stockholm, Sweden; ^2^Center for Clinical Research Sörmland, Uppsala University, Eskilstuna, Sweden; ^3^Karolinska University Hospital, Stockholm, Sweden; ^4^Danderyd Hospital, Stockholm, Sweden

**Keywords:** cerebral palsy, spasticity, adult, pain, randomized controlled trial, Botulinum Toxin-A

## Abstract

**Objective:** To test if botulinum toxin-A (BoNT-A) is effective in reducing chronic muscle-related pain in adults with spastic cerebral palsy (CP), as compared to placebo.

**Design:** A single-center, double-blind, parallel, randomized placebo-controlled trial. The design included an interim analysis to allow for confirmatory analysis, as well as pilot study outcomes.

**Setting:** Tertiary university hospital.

**Participants:** Adults with spastic CP and chronic pain associated with spastic muscle(s).

**Intervention:** Treatment was one session of electromyographically guided intramuscular injections of either BoNT-A or placebo normosaline.

**Main Study Outcomes:** The primary outcome was the proportion who achieved a reduction of pain intensity of two or more steps on the Numerical Rating Scale 6 weeks after treatment.

**Results:** Fifty individuals were screened for eligibility, of whom 16 were included (10 female, 6 male, mean age = 32 years, SD = 13.3 years). The randomization yielded eight participants per treatment arm, and all completed the study as randomized. The study was stopped at the interim analysis due to a low probability, under a preset threshold, of a positive primary outcome. Four individuals were treatment responders in the BoNT-A group for the primary outcome compared to five responders in the placebo group (*p* = 1.000). Adverse events were mild to moderate. In exploratory analysis, the BoNT-A group had a trend of continuing reduction of pain at the last follow-up, after the primary endpoint.

**Conclusions:** This study did not find evidence that BoNT-A was superior to placebo at the desired effect size (number needed to treat of 2.5) at 6 weeks after treatment.

**Trial registration:**ClinicalTrials.gov: NCT02434549

## Strengths and Limitations of The Study

- Researcher-initiated and academically funded, randomized, placebo-controlled double-blind clinical trial.
- The trial was stopped at the interim analysis due to a low probability of a positive primary outcome (i.e., stopped for futility) resulting in a small sample size.
- This study presents the first data on effect sizes in pain treatment trials in adults with CP.

## Introduction

One particularly important health issue in adults with cerebral palsy (CP) is pain. Pain, often chronic in character, is reported to affect up to 76% of adults with CP (1). Despite the high prevalence of pain, very little, if any, is known on how to address this issue. To the best of our knowledge, no clinical trial has been published where pain reduction has been the primary outcome in adults with CP and in only one case as an exploratory variable (2). The need for studies primarily focused on pain management in CP has been requested as a top research priority by individuals with CP and the involved community (3).

The etiology of the pain in CP is incompletely understood and, most likely, diverse in nature (4). Common clinical explanatory causes include arthropathy, postsurgical pain, neuropathic pain, and muscle-tone abnormalities. Spasticity, a commonly proposed causative factor (4, 5), is present in 9 of 10 individuals with CP (6). Spasticity is characterized by a velocity-dependent resistance of a muscle to stretch (7) and could, hypothetically, cause mechanical stresses on musculoskeletal structures with secondary development of chronic pain. Although spasticity is a quite frequent finding in neurological disorders, many aspects of spasticity differ because of the etiology. These include, but are not limited to, the onset of spasticity after an event, the development or change of spasticity over time, and the possible neuroradiological findings that are considered to correlate with the spasticity. Thus, findings from studies on spasticity reduction in multiple sclerosis or traumatic brain injury, for example, cannot be directly extrapolated onto CP.

For over 30 years, botulinum toxin-A (BoNT-A) has been used extensively to treat spasticity in CP owing to its muscle-relaxing effects (8). After intramuscular injection, BoNT-A acts by blocking the presynaptic release of acetylcholine at the neuromuscular junction causing dose-dependent levels of muscle paralysis (9).

Overall, common indications for BoNT-A have been disorders characterized by muscle hyperactivity such as spasticity and dystonia. There is, however, also high-level evidence for its efficacy in several pain conditions not associated with increased muscle tone including chronic migraine, postherpetic neuralgia, and trigeminal neuralgia (10, 11). Proposed mechanisms of analgesia include altered neurotransmitter release of sensory nerves and central modulatory effects (9–11).

There is clinical experience that some children and adults with CP and pain related to spastic muscle can respond to BoNT-A treatment (12). The evidence base is, however, minimal (13). In the literature, there are two trials in children with CP, comparing BoNT-A with placebo, with pain reduction as the primary outcome. Barwood et al. reported significant advantages in pain reduction, the need for other analgesics, and duration of hospital stay in children with spastic CP who received BoNT-A before soft-tissue hip surgery (14). Will et al., however, reported no differences in pain reduction, quality of life, need for other analgesics, or hospital stay in children with spastic CP who received BoNT-A before skeletal hip surgery (15). Overall, there is a scarcity of studies systematically evaluating interventions for pain in CP including the use of BoNT-A (13).

This study was designed in light of the burden that pain poses to individuals with CP and the lack of evidence-based interventions, as well as the theoretical therapeutic potential of BoNT-A. Given the limited existing information on expected efficacy, the trial incorporated an interim analysis. The sole purpose of this midtrial analysis was to determine whether the trial was likely to fail given its preset parameters. This allowed the study to serve a dual purpose. If the interim analysis recommended continuation of the trial, it would fulfill its confirmatory purpose; if the recommendations was to stop, the trial would provide pilot study data for future trials without subjecting unnecessarily many participants to inclusion in a futile trial.

## Methods

### Design

This was an academically initiated and funded single-center, double-blind, parallel, randomized, placebo-controlled trial with an even randomization ratio. The study was approved by the Stockholm Regional Ethical Review Board (2015/271-31/2) and the Swedish Medical Products Agency (2015-000095-10) and was preregistered at ClinicalTrials.gov (NCT02434549).

### Study Participants and Setting

The study was conducted at a tertiary referral center in Stockholm, Sweden. Adults with CP were recruited through referrals from clinicians at all care levels in Stockholm and adjacent counties and through public advertisements in newspapers, on patient organization websites and in medical facilities. Inclusion criteria were age ≥18 years, spastic type of CP according to Surveillance of Cerebral Palsy in Europe guidelines (16), chronic pain related to spastic muscle [duration ≥3 months, intensity ≥3 on Numerical Rating Scale (NRS)], and signed informed consent. Exclusion criteria were hypersensitivity to BoNT-A, pregnancy, breastfeeding, treatment with BoNT-A within the last 5 months, changes in muscle-tone–altering medications within the last 2 weeks, clearly degenerative pain mechanisms, and/or intellectual disability or communication impairments that disabled the individual from independently giving informed consent.

### Study Timeline

The screening and baseline visit and follow-ups were performed by one team (D.J., K.L.) at the Karolinska University Hospital, Stockholm. Each participant was interviewed and assessed for muscle-related pain and examined for regional spastic muscles to be targeted for injection. The treatment was given between 0 and 21 days after the baseline visit at Danderyd Hospital, Stockholm, by another team (K.K., B.M.R.). A telephone contact was made 1 week after treatment (D.J., K.L.), and at 6 weeks, there was a return visit to the team where primary and secondary outcomes were assessed (D.J., K.L.). A final telephone follow-up occurred 10 weeks after the treatment (D.J., K.L.). See Table 1 for a summary of study time points.

**Table 1:** Overview of study time points.

**Time point**	**Baseline**	**Treatment**	**Posttreatment**		
			**1 week**	**6 weeks**	**10 weeks**
**Location**	**Karolinska hospital**	**Danderyd hospital**	**Telephone**	**Karolinska hospital**	**Telephone**
**Team**	**D.J., K.L.**	**K.K., B.M.R.**	**D.J., K.L.**	**D.J., K.L.**	**D.J., K.L.**
**Variable**					
NRS	x		x	x	x
Analgesics	x			x	x
BPI	x			x	x
SF-36v2	x			x	
PGIC				x	
FSS	x			x	
MAS	x			x	
ROM	x			x	
AE	x	x	x	x	x

*NRS, Numerical Rating Scale (of pain intensity); BPI, Brief Pain Inventory–Short Form; SF-36v2, Short Form 36 version 2 (Health-Related Quality of Life); PGIC, Patient Global Impression of Change scale; FSS, Fatigue Severity Scale; MAS, Modified Ashworth Scale according to Bohannon and Smith (spasticity); ROM, range of motion; AE, adverse events*.

### Intervention

The treatment consisted of one session of electromyographically guided intramuscular injections of either BoNT-A (abobotulinumtoxin-A, Dysport®, 100 U/mL, up to a maximal total dose of 1,500 U), or normosaline in the corresponding volume (placebo). For treatment details, see Table 2, and for injected muscles, see Table 3.

**Table 2:** Participant characteristics and treatment details.

	**Group allocation**	
	**BoNT-A**	**Placebo**
**Participant characteristics**		
Participants, *n*	8	8
Age, median (range), years	24 (18–60)	33 (21–50)
Female sex, *n* (%)	5 (63%)	5 (63%)
Subtype of spastic CP, bilateral/unilateral	4/4	7/1
GMFCS levels, I–II/III–IV	6/2	3/5
BoNT-A treatment ≤ 12 months, *n*	1	2
NRS baseline, median (range)	5 (4–7)	5 (4–9)
Pain interference baseline, ^a^mean (SD)	4.7 (1.6)	5.5 (2.6)
Opioid treatment at baseline, *n*	1	1
**Treatment**		
Target, LE/LE and UE	7/1	8/0
No. of muscles, median (range)	2.5 (1–4)	4 (1–4)
No. of injections, median (range)	13 (8–24)	15 (8–24)
Dose, ^b^median (range), U	920 (660–1,500)	—
Dose, ^b^median (range), mL	9.2 (6.6–15)	10.2 (4–13.9)

a *As assessed on the BPI*.

b *Units (U) of Dysport®, 100 U/mL, per participant, as actually given in the BoNT-A group; given as the equivalent volume of normosaline only in the placebo group*.

*CP, cerebral palsy; GMFCS, Gross Motor Function Classification System (17); BoNT-A, botulinum toxin-A; NRS, Numerical Rating Scale of pain intensity; SD, standard deviation; BPI, Brief Pain Inventory–Short Form; LE, lower extremity; UE, upper extremity*.

**Table 3:** Injected muscles per participant, with treatment allocation.

**Participant**	**Group allocation**	
	**BoNT-A**	**Placebo**
1	Adductor magnus R Adductor brevis R Adductor longus R Medial hamstrings R	
2		Adductor brevis R and L Medial hamstrings R and L
3		Gastrosoleus R
4	Gastrosoleus R	
5	Medial hamstrings R and L Rectus femoris R and L	
6		Adductor magnus R Adductor brevis R Adductor longus R Rectus femoris R
7	Gastrosoleus L	
8	Gastrosoleus L	
9		Medial hamstrings R and L Gastrocnemius R and L
10		Adductor magnus R and L Adductor brevis R and L Adductor long R and L Medial hamstrings R and L
11		Medial hamstrings L Lateral hamstring L Gastrocnemius L
12	Medial hamstrings R and L Gastrocnemius R and L	
13	Gastrosoleus R and L	
14		Medial hamstrings R and L Gastrocnemius R and L
15		Medial hamstrings R and L
16	Medial hamstrings R Gastrosoleus R Biceps brachii R	

### Outcomes

Selection of outcomes adhered to guidelines from IMMPACT (Initiative on Methods, Measurement and Pain Assessment in Clinical Trials) (18).

The primary outcome was the proportion of treatment responders, defined as a reduction of pain intensity of two or more steps on the NRS, at 6 weeks after treatment, compared to baseline.

Secondary outcomes were

1) Categories of change in the use of analgesic treatments compared to baseline. This was defined as either increased, unchanged, or decreased at 6 weeks after treatment.
2) The proportion of responders derived as a reduction in the mean pain interference score of ≥1 on the Brief Pain Inventory (19) at 6 weeks after treatment. The pain interference items capture the consequences of pain on general activities, mood, walking ability, normal work, relations with other people, sleep, and enjoyment of life.

Exploratory outcomes were self-reported health status using the Short Form-36 version 2 (SF-36v2) (20), participant overall satisfaction with the treatment using the Patient Global Impression of Change (PGIC) scale (21), and severity of mental fatigue (e.g., lacking energy and/or feeling of tiredness not restituted by rest) using the Fatigue Severity Scale (FSS) (22). Spasticity was assessed using the Modified Ashworth Scale according to Bohannon and Smith (MAS) (23) and passive joint range of motion (ROM) measured with a goniometer in standardized positions. There were no changes in outcomes or eligibility criteria after trial commencement. See Table 1 for time points and variables. Any adverse events were recorded continuously.

### Sample Size and Interim Analysis

There were no prior data on the expected efficacy of pain reduction in adults with CP. The sample size was calculated with the goal of detecting a proportion of 70% treatment responders in the group receiving BoNT-A and 30% treatment responders in the placebo group. This corresponds to a number needed to treat (NNT) efficacy of 2.5. Statistical power (1 – β) was set at 0.8 and α at 0.05. This yielded a sample size of *n* = 42 (*n* = 21 per group).

The interim analysis, performed by an independent Data Monitoring Committee, was included in the protocol with evaluation of one criterion: *stop for futility* (defined as <20% probability of showing treatment superiority). The rationale for the interim analysis was (1) very limited prior data on expected efficacy and (2) limited data on expected inclusion rate. This design allowed the study to fulfill two different purposes: if the study was not stopped at the interim analysis, it would fulfill its confirmatory design of accepting or rejecting efficacy of BoNT-A or, if the study was stopped at the interim analysis, it would be a pilot study for future confirmatory studies.

### Randomization, Treatment Allocation, and Blinding

The study statistician prepared a computer-generated treatment allocation randomization list with random block sizes. Participants were entered on the list sequentially at enrollment and identified through their sequential study ID. The study nurse preparing the injections was the only individual (except for the statistician) with access to this list. The study nurse was not involved in any other part of patient care or data collection. At the treatment session, the study nurse prepared the syringes, marked these with study ID only, and brought these to the physician positioned in an adjacent room. Reconstituted BoNT-A is a clear, water-like solution indistinguishable from normosaline on inspection. The treatments were performed in an identical fashion regardless of allocation. Treatment allocation was altogether double-blind: allocation was unknown to study participants, to the screening and evaluating team, and to the team performing the treatment.

### Statistical Analyses

The primary and secondary outcomes were analyzed using Fisher exact test of proportions on the categories of response by treatment. Exploratory analysis of magnitude of pain reduction by treatment arms was tested using independent *t*-test with unequal variance. The independent *t*-test was also used to test pretreatment and posttreatment differences in FSS and SF-36v2, whereas spasticity (MAS) and PGIC were tested using Wilcoxon rank-sum test. Differences in proportion of significant improvement in ROM (>10 degrees) were tested using Fisher exact test. Adverse events were prepared descriptively. The significance level was set at *p* ≤ 0.05.

## Results

**Figure 1:**
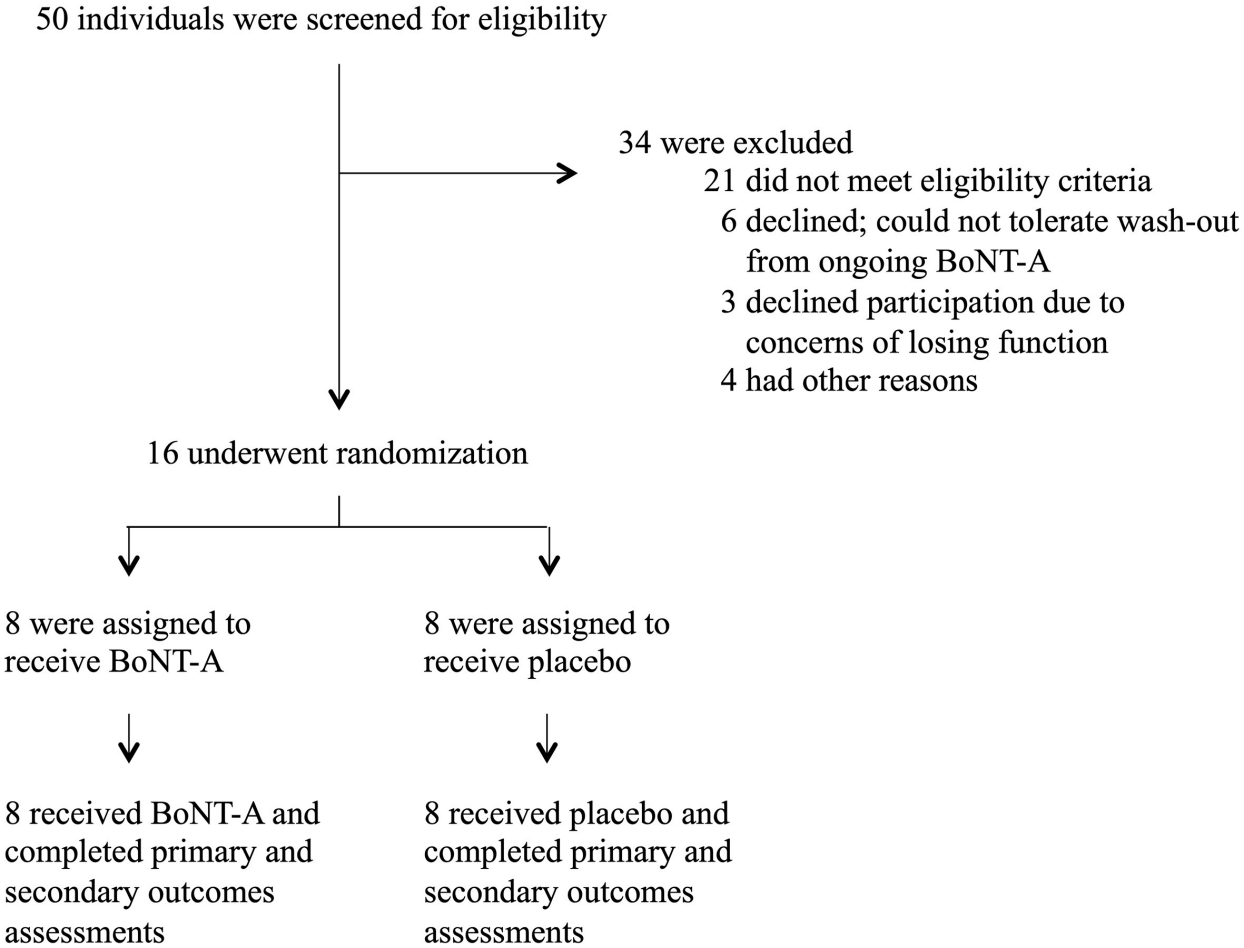
Study patient flowchart. BoNT-A, botulinum toxin-A.

A total of 50 individuals were screened for eligibility (Figure 1). Sixteen participants were included and randomized (10 female and 6 male participants, mean age = 32 years, SD = 13.3 years), with eight participants in each treatment arm. The most common causes for exclusion were that the individual presented with pain that appeared only infrequently (not daily as per the definition of chronic pain) or that the pain was unrelated to regional spasticity. Other common pain types during the screening process were neuropathic pain and joint pain. The inclusion process is illustrated in Figure 1. Baseline characteristics are shown in Table 2. There were no significant differences in age, sex, pain intensity, or any other baseline characteristic between groups. Inclusion began in September 2015 and was stopped in October 2018. The study was terminated at the interim analysis due to futility of the primary outcome. All randomized participants received the intended treatment and were assessed for the primary and secondary outcomes.

**Figure 2:**
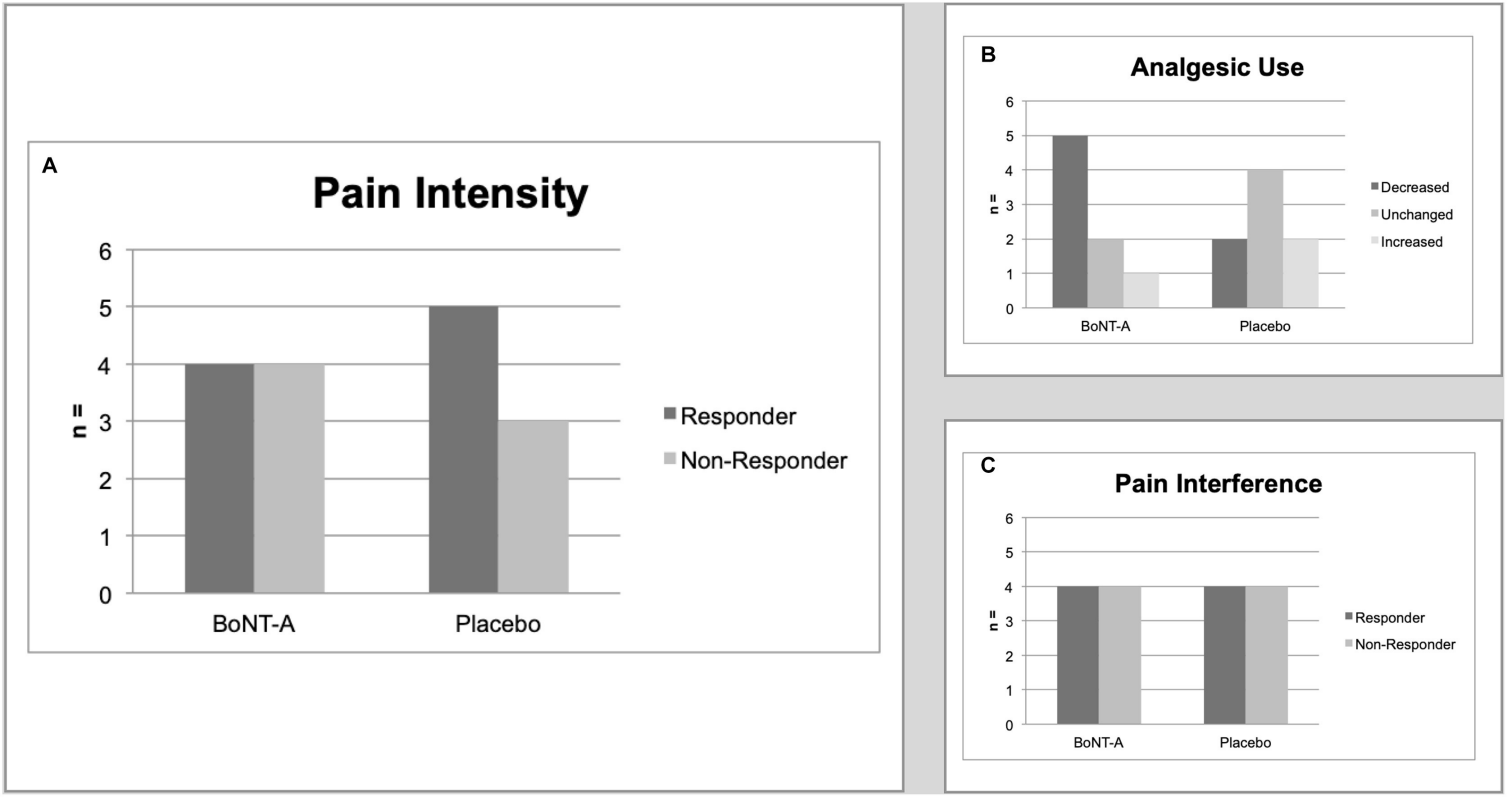
Main study outcomes. **(A)** Primary outcome. The number of treatment responders (defined as a reduction of ≥2 scale steps on the NRS) at 6 weeks after treatment, by treatment group. Test of proportions, *p* = 1.000. **(B)** Secondary outcome. The number of treatment responders (defined as change in categories of analgesics use) at 6 weeks after treatment, by treatment group. Test of proportions *p* = 0.429. **(C)** Secondary outcome. The number of treatment responders (defined as a reduction of mean interference score of ≥1 on the BPI) at 6 weeks after treatment, by treatment group. Test of proportions *p* = 1.000. BPI, Brief Pain Inventory; NRS, Numerical Rating Scale.

There were four treatment responders and four non-responders in the group receiving BoNT-A for the primary outcome of pain intensity at 6 weeks after treatment as compared to five treatment responders and three non-responders in the placebo group (test of proportions *p* = 1.000) (Figure 2).

In the group receiving BoNT-A, five participants had decreased their concomitant analgesic use at 6 weeks after treatment, two were unchanged, and one had an increased use. In comparison, in the placebo group two participants had decreased their analgesic use, four were unchanged and two had increased their use (*p* = 0.429) (Figure 2).

Four participants in each treatment arm reported a reduction of ≥1 score points on mean pain interference (*p* = 1.000) (Figure 2).

### Exploratory Analyses

**Figure 3:**
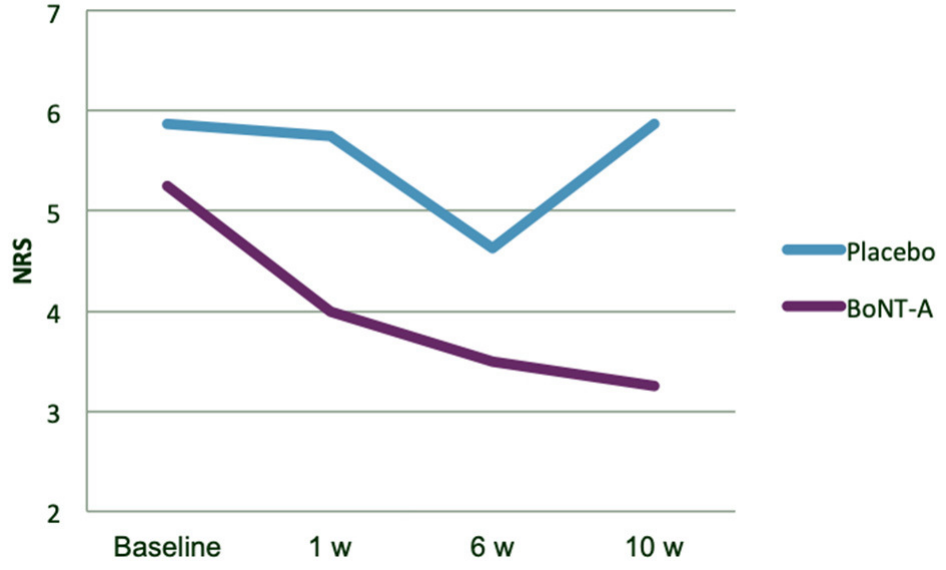
Exploratory analysis of mean pain intensity, by treatment group. BoNT-A, botulinum toxin-A; NRS, Numerical Rating Scale.

**Figure 4:**
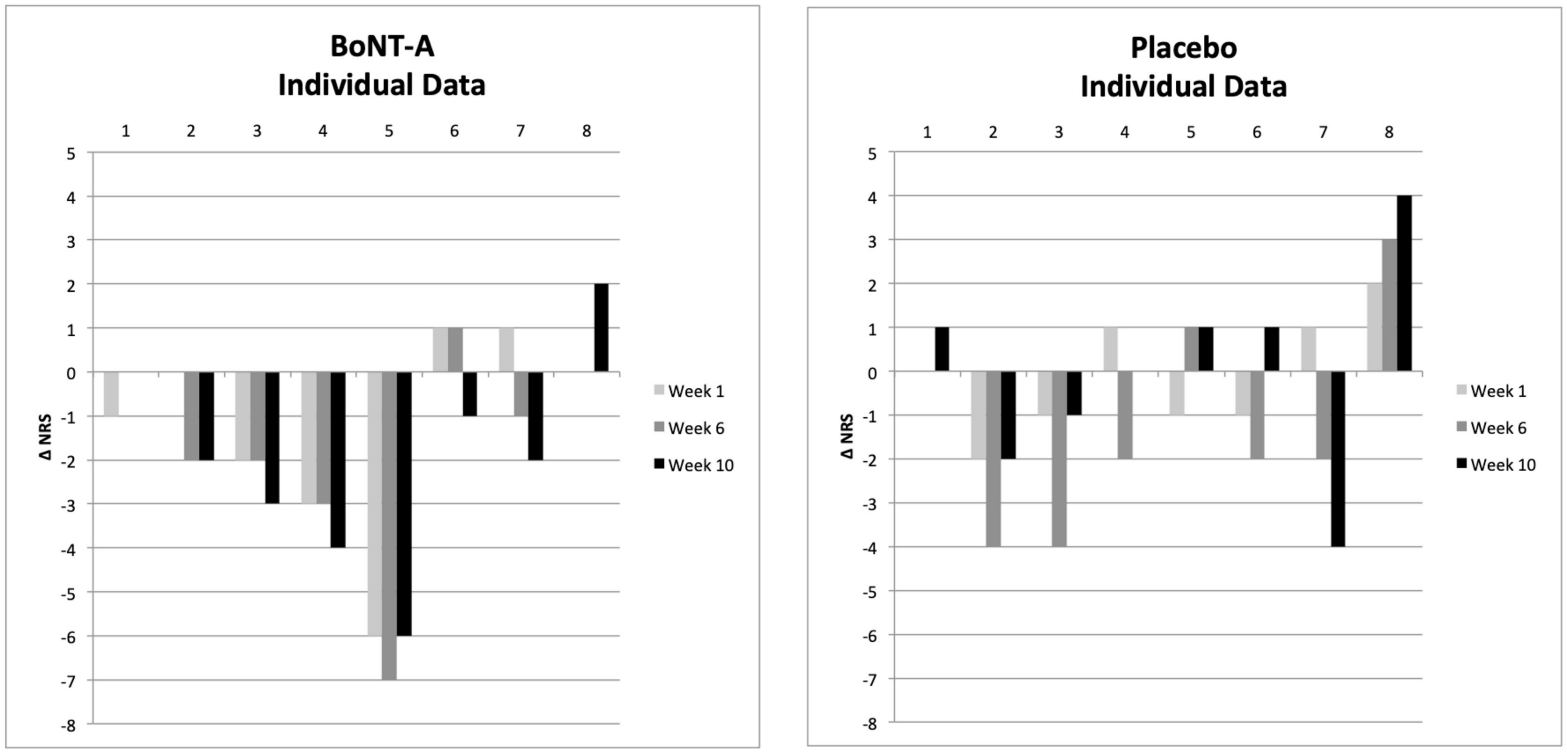
Exploratory analysis of individual patient pain intensity, by treatment group. Δ NRS represents change in pain intensity as compared to baseline. BoNT-A, botulinum toxin-A; NRS, Numerical Rating Scale.

The magnitude of change in pain intensity was subject to *post-hoc* analysis (Figures 3, 4). There was a trend for a continuing reduction of pain intensity in the group receiving BoNT-A not seen in the placebo group (Figure 3). At 10 weeks after treatment, the mean and median pain reduction was 2.0 NRS scale steps in the BoNT-A group and 0.0 NRS scale steps in the placebo group (difference = −2.0, 95% CI = −0.60 to 4.60, *p* = 0.121). Data on individual pain intensity over time are shown in Figure 4. There were no significant group differences in pain interference (as assessed with the BPI) at 10 weeks after treatment (data not shown).

There were no significant differences when comparing treatment differences on PGIC, FSS, or SF-36v2 physical component score, mental component score, or bodily pain (Table 4).

**Table 4:** Exploratory analyses of patients' global impression of change, fatigue severity, and self-reported physical and mental health at 6 weeks after treatment.

	**BoNT-A *n* = 8**	**Placebo *n* = 8**	**Statistical test *p***
**PGIC**			0.499
Very much improved	0	0	
Much improved	2	1	
Minimally improved	4	4	
Unchanged	1	1	
Minimally worsened	0	1	
Much worsened	1	1	
Very much worsened	0	0	
**FSS**			
Baseline, mean (SD)	4.0 (1.2)	5.1 (1.6)	
Difference after treatment, mean (SE)	+0.2 (0.4)	−0.2 (0.2)	0.401
**SF-36v2**			
**PCS**			
Baseline, mean (SD)	41.2 (7.4)	36.9 (6.5)	
Difference after treatment, mean (SE)	+4.1 (2.5)	+2.4 (2.0)	0.602
**MCS**			
Baseline, mean (SD)	46.6 (8.6)	39.8 (16.1)	
Difference after treatment, mean (SE)	+3.4 (4.6)	+4.6 (2.8)	0.832
**Bodily pain**			
Baseline, mean (SD)	37.4 (6.3)	34.1 (8.5)	
Difference after treatment, mean (SE)	+8.2 (3.6)	+3.2 (3.5)	0.329

*SF-36v2 results are norm-based scores, normalized to center on 50 for the referral population, with 1 standard deviation = 10. For SF-36v2 norm-based scores, higher scores are better, and lower scores are worse, e.g., an increase in bodily pain scores signifies an improvement*.

*BoNT-A, botulinum toxin-A; FSS, Fatigue Severity Scale; MCS, mental component score; PCS, physical component score; PGIC, Patient Global Impression of Change Scale; SD, standard deviation; SE, standard error; SF-36v2, Short Form 36 version 2*.

Muscle spasticity was reduced one or more scale steps in 80% of muscles treated with BoNT-A and in 50% of muscles treated with placebo (Table 5). There were no significant group differences in changes in MAS or ROM. There was no apparent correlation (Spearman correlation coefficient = 0.11, *p* = 0.709) between being a treatment responder at the primary endpoint and having a significant reduction of spasticity (MAS reduction ≥1).

**Table 5:** Differences in spasticity (MAS) and joint range of motion (ROM) after treatment, compared to baseline.

	**BoNT-A**	**Placebo**	**Statistical test *p***
**MAS (scale steps)**			0.078
Min	0	0	
Max	−2	−3	
Median	−2	−0.5	
Mode	−2	0	
Proportion ≥-1	80%	50%	
Proportion ≥-2	60%	30%	
**ROM (degrees)**			
Proportion ≥+10	42%	38%	0.788

*The results refer to injected muscles, and ROMs associated with the injected muscles. For MAS, results are presented both as treatment group median, minimum, maximum, and mode difference and the proportion of muscles in each treatment group achieving more than 1 and 2, scale steps reduction on the MAS scale. For ROM, results represent the proportion of ROMs where an improvement of 10 degrees or more was seen, per treatment group*.

*MAS, Modified Ashworth Scale according to Bohannon and Smith; ROM, (joint) range of motion; BoNT-A, botulinum toxin-A; Min, minimum value; Max, maximal value*.

### Adverse Events

There was one serious adverse event in which a participant in the placebo group was diagnosed with lymphoma during the study period, a diagnosis that was interpreted as unrelated to study treatments or events. Study-related adverse events were mild to moderate: the most common adverse event was mild pain and discomfort during and immediately after the intramuscular injections, which was reported by five of eight participants (75%) in the BoNT-A group and seven of eight (88%) in the placebo group. Two participants (38%) in the BoNT-A group reported transient focal weakness in treated muscles, which in one case briefly interfered with activities of daily living (moderate severity), whereas no such event occurred in the placebo group.

## Discussion

This study is the first randomized controlled trial aimed at reducing pain in adults with CP. As such, the results are of significant value for future interventional studies within this largely unexplored field.

As a general reminder, pain is a complex phenomenon with an often multifactorial background. Pain does not become less complex when it is combined with a childhood-onset disability such as CP. Establishing anchoring points for “zero” pain on the NRS can be difficult if the individual has had lifelong pain and discomfort. Likewise, setting inclusion cutoff values for pain intensity (or pain interference) can be difficult, as adults with CP and pain could have adapted their lives to minimize painful activities. Notwithstanding these difficulties, it is important to find effective treatments through randomized trials.

The primary outcome of responder analysis at 6 weeks after treatment failed to show a difference between BoNT-A and placebo at the effect size corresponding to an NNT of 2.5. This is a reasonable effect size to strive for when comparing with the efficacy of common, less expensive, non-opioid analgesics in other disorders (24). The results obtained do not exclude that smaller effect sizes are possible at 6 weeks after treatment. More interesting is the fact that the results indicate that the (possible) analgesic effect of BoNT-A comes later than what was initially assumed. The mean pain intensity in the BoNT-A group continued to trend downward at the last follow-up (10 weeks). When BoNT-A is used to treat spasticity in CP, the onset of therapeutic effect is within a few days, peaking ~4 weeks after injection, typically with a sustained effect for 3–5 months (8, 25). This was the main basis for the timing of the primary outcome at 6 weeks after injection. Will et al. had the same preconception (primary outcome at 6 weeks) in their recently published trial on preoperative BoNT-A for bony surgery–related pain in children with CP, which failed to show superiority compared to placebo (15). Our results lead to a hypothesis that the supposed effect of BoNT-A could be through other mechanisms than spasticity reduction. Pain relief does not necessarily coincide with muscle relaxation when BoNT-A is used for established pain indications (26). For example, compared to placebo, the effect of BoNT-A was more pronounced 3 months after a single injection in one of the first trials on migraine (27). Other than muscle relaxation, modulation of peripheral neurotransmitter release, anti-inflammation, and central nervous system modulatory effects have been proposed as alternative modes of action in BoNT-A–mediated pain relief (9, 26). These modes of action could potentially modulate painful secondary musculoskeletal effects associated with spasticity, mentioned in the introduction. If these are the pathways in play in spastic muscle-related pain in CP, then future research should incorporate longer follow-up with later endpoints. Additionally, it is possible that the placebo effect, which is considerably large in chronic pain trials (28), wanes off before the pharmacological effects of BoNT-A do, also prompting longer follow-up. The placebo effect was apparent on MAS and ROM, where improvements could be seen in both groups with a slight, but not statistically significant, added effect in the BoNT-A group. At the primary endpoint (6 weeks), 80% of the treated muscles in the BoNT-A group showed a significant reduction of spasticity compared to 50% in the placebo group. The distribution of treatment responders at this time point (in slight favor of the placebo group) further puts into question the role of pure spasticity reduction in pain relief in this setting.

Another finding from this study is that certainly not all adults with CP and chronic pain who were screened for eligibility had muscle-related pain associated with spastic muscle. This was the main reason for non-eligibility in the screening process. Other pain modalities were also seen such as intra-articular pain and neuropathic pain, which indicate the need for further investigations on the epidemiology of, and mechanisms behind, chronic pain in adults with CP.

Adverse events were generally mild and related to the injection procedure. Two participants in the BoNT-A group experienced transient focal weakness, a well-recognized possible side effect of this drug.

Study limitations include study size, a consequence of the termination of the trial at the interim analysis. Another limitation is that there are few or no instruments and questionnaires specific to adults with CP. Some items in the generic questionnaires are poorly suited for individuals with childhood-onset disability. This has the potential of causing non-differential misclassifications on those items, which could make the study results less accurate. Development of condition-specific outcome measures would be of value for future studies. Also, the validity of the often-used MAS as a measurement of spasticity has been questioned (29).

## Conclusions

This study was stopped at the interim analysis as there were no indications that BoNT-A was more effective than placebo in reducing chronic muscle-related pain in adults with spastic CP at 6 weeks after treatment. Further trials of longer duration are nevertheless warranted, as the BoNT-A group displayed a trend of continuous pain reduction at the last follow-up. This study can be used as a pilot study in the design of chronic pain trials in adults with CP.

## Data Availability Statement

The raw data supporting the conclusions of this article will be made available by the authors, without undue reservation.

## Ethics Statement

The studies involving human participants were reviewed and approved by Stockholm Regional Ethical Review Board. The patients/participants provided their written informed consent to participate in this study.

## Author Contributions

DJ coordinated the study, drafted the manuscript, and contributed to the design and the data collection. KL contributed to the design, the data collection, reviewed, and approved the final manuscript for publication. KK and B-MR contributed to the design, the data collection, and approved the final manuscript for publication. KT conceptualized the design and had senior responsibility for the study, reviewed, and approved the final manuscript for publication. No other individual fulfilled criteria for authorship. All authors contributed to the article and approved the submitted version.

## Conflict of Interest

KK has received speakers' honorarium from Ipsen and Allergan. The other authors have no conflicts of interest to report.

## Publisher's Note

All claims expressed in this article are solely those of the authors and do not necessarily represent those of their affiliated organizations, or those of the publisher, the editors and the reviewers. Any product that may be evaluated in this article, or claim that may be made by its manufacturer, is not guaranteed or endorsed by the publisher.
